# Methods for Involving People With Dementia in Health Policy and Guideline Development: A Scoping Review

**DOI:** 10.1111/hex.70250

**Published:** 2025-04-03

**Authors:** Felix Bühler, Jennifer Geyer, Gabriele Meyer, Anja Bieber

**Affiliations:** ^1^ Institute of Health and Nursing Science Medical Faculty of Martin Luther University Halle‐Wittenberg, University Medicine Halle Halle (Saale) Germany

**Keywords:** dementia, guideline, health policy, patient and public involvement, public engagement, scoping review

## Abstract

**Introduction:**

Patient and public involvement (PPI) is considered part of best‐practice for health care delivery, research and policy. However, people with dementia are frequently excluded from PPI initiatives. While recent studies have investigated PPI of people with dementia in research, little is known about their involvement at the macro‐level of care, that is, in health policy and guideline development. This scoping review maps the evidence on PPI of people with dementia at the macro‐level of care, focusing on the methods, outcomes and mechanisms of involvement.

**Methods:**

We systematically searched MEDLINE via PubMed, CINAHL, the Cochrane Library and GeroLit. Additionally, we performed forward and backward citation searching, manually tracked individual references, searched abstract books and yearbooks, and contacted authors of included reports to seek additional references. We analysed each method's mechanisms of involvement to assess whether measures were taken to maximise effective information transfer.

**Results:**

We included 43 reports and identified 35 involvement methods, which we structured into six categories: ‘focus groups and interviews’, ‘surveys and questionnaires’, ‘public events’, ‘meetings with decision‐makers’, ‘serving as members of working groups’, and ‘multiple‐step methods’. Most of the involvement took the form of consultations during the early stages of policy or guideline development. All involvement methods required verbal communication skills, almost all of the participants had mild dementia. We found that most reports did not clearly state the involvement outcomes. While a majority of methods had some facilitation in place to elicit information from participating people with dementia, only nine methods used a structured aggregation to synthesise participants' contributions into a joint statement.

**Conclusion:**

We found limited evidence of dementia‐adapted involvement. Future research might focus on exploring the mechanisms of involvement to adapt methods to specific target groups, such as people with impaired verbal communication or advanced dementia. We recommend using existing guidance to report PPI initiatives, as the reporting was often incomplete, which limits reproducibility.

**Patient or Public Contribution:**

We discussed both our study protocol and our results with a working group of people with dementia, who provided valuable insight for our data interpretation. Our findings can serve such groups for future consultations.

## 1 Introduction

The perspectives of patients and health service users have gained impact in healthcare in recent years. With patient‐centredness becoming a key tenet of healthcare, patient and public involvement (PPI) has become a widely accepted objective for healthcare delivery, research, and policy [1, 2]. Involving patients and service users is considered best practice, with researchers, patient organisations, funders and regulatory authorities endorsing PPI in healthcare [2, 3].

However, despite these unanimous endorsements, people with conditions affecting communication or cognition, such as dementia, have remained largely excluded from PPI, based on the assumption that they are unable to contribute [4, 5]. Recruitment and continuous involvement of people with dementia have emerged as PPI barriers, especially in view of the progressive nature of dementia and disease‐specific impairments of memory, language or attention [5, 6]. As most PPI activities appear to be dependent on cognitive abilities, people with advanced dementia are the least likely to be involved in PPI [7].

Recent studies have investigated the involvement of people with dementia in research [6, 8], and focused on the methods used to involve them [4, 9, 10, 11]. This focus on methods reflects the recognition that those commissioning PPI should accommodate participants by selecting suitable methods of involvement or adapting existing methods to meet their target groups' specific needs. For instance, research methods have been tailored to people with dementia by visualising interview questions, allowing the participant to decide the time and date of data collection, or using familiar objects and life story books as interview cues [4].

Despite these recent advances, little is known about the methods used to involve people with dementia at the macro‐level of care, that is, in health policy, legislation and guideline development [12]. Patient and public involvement at this level is assumed to contribute to patient‐centredness in policies and guidelines, improved health outcomes and more democratic decisions [13, 14]. However, it is unclear to what extent people with dementia have been involved in policymaking and guideline development to date, what methods have been used to involve them, and what outcomes their involvement has led to.

Given the overall objective of PPI at the micro‐, meso‐ and macro‐level of care and the recognised challenges of involving people with dementia, a synthesis of evidence on target group‐specific involvement methods is needed. We therefore conducted a scoping review to map the available evidence on methods used to involve people with dementia in health policy and guideline development. Our review addresses the following research questions:

- Which methods are used to involve people with dementia in health policy and guideline development, and what are the theoretical underpinnings of these methods?
- Which outcomes have resulted from using these methods?
- Which barriers and facilitators are reported for these methods?
  In view of the particular challenges of involving people with dementia, we additionally aimed to examine any measures taken to maximise the transfer of relevant information in these involvement methods. Therefore, we sought to analyse each method's underlying involvement mechanisms using the public engagement typology by Rowe and Frewer [15]. Consequently, we added the following question:
- Which underlying involvement mechanisms are these methods based on?

## 2 Materials and Methods

### 2.1 Design

Our scoping review was guided by the methodology proposed by Levac et al. [16] and the Joanna Briggs Institute (JBI) [17]. We chose the scoping approach as we expected to find a heterogeneous body of evidence, and aimed to give an overview of all available evidence on this topic [17]. Before commencing the study, we registered the study protocol on OSF [18]. Reporting adheres to the PRISMA‐ScR extension [19].

### 2.2 Eligibility Criteria

This review aimed at methods used to involve people with dementia at a macro‐level of care [12], that is, policymaking for health or social care, or development of health or social care guidelines. We included any empirical or nonempirical (i.e., conceptual or theoretical) reference describing methods to involve people with dementia in these activities, regardless of whether the involvement or the methods were primary outcomes of the reports or merely secondary findings. Study reports, editorials, protocols, book chapters, conference abstracts and grey literature (preprints, unpublished reports) in English or German were considered eligible.

We excluded references describing the involvement of people with dementia in research, even where guidelines were mentioned as an output, as evidence on the involvement in research is already available [6, 8]. Furthermore, we excluded reports on the involvement at individual or service levels of care (i.e., micro‐ or meso‐level), as well as comments and opinion‐based articles without pertinent description of involvement methods. Health or social care guidelines were considered eligible if they (i), were informed by a systematic synthesis of evidence, (ii) aimed at care professionals rather than patients or relatives alone, and (iii) had at least a regional scope of application, as opposed to guidelines for individual care facilities or organisations. We did not limit our search to clinical topics, guidelines from social care were eligible if they met the criteria above.

### 2.3 Information Sources

We searched MEDLINE (via PubMed), CINAHL, the Cochrane Library, and GeroLit on November 28 and 29, 2023. Additionally, we performed hand searches in the OECD Library, in abstract books from the Alzheimer Europe and Alzheimer's Disease International conferences (2014–2023), and in Alzheimer Europe's yearbooks (2014–2023). Based on all included references, we performed forward and backward citation searching via Web of Science on June 12, 2024. For references not indexed in the Web of Science, we manually tracked potentially relevant references. Finally, where information was incomplete, we contacted the authors to make enquiries and ask about additional relevant references.

### 2.4 Search Strategy

FB drafted the PubMed search strategy based on the Population‐Concept‐Context (PCC) mnemonic by identifying potential search terms via preliminary searches [17]. The strategy consisted of title and abstract search terms and MeSH terms, combined with Boolean Operators. All co‐authors and one additional researcher with experience of systematic reviews subsequently revised the strategy. We added a fourth search component specifying the concept component, as the relevant search terms (participation, involvement etc.) are used in various contexts and, without specification, resulted in many irrelevant references. FB manually translated the finalised Medline via PudMed search strategy to the other databases (search strategies are available in Data S1).

### 2.5 Data Management and Study Selection

FB performed the database searches, citation searching, deduplication of references and contacted authors to obtain additional information. FB and JG performed the study selection by first screening titles and abstracts, then by assessing full texts, using the blinded screening mode of the online application *Rayyan*. A pilot phase of 50 references preceded the screening process to assess consistency and agreement. We applied the same screening process for potentially relevant references from the hand searches identified by a third person. Conflicting decisions were solved via discussion.

### 2.6 Data Charting and Analysis

We did not perform a critical appraisal as only a minority of included references were empirical study reports. For data extraction, we drafted a template based on our research questions. FB and JG pilot tested the template with five references to assess applicability and agreement of charting. Following minor adjustments to the template, FB and JG independently charted and mutually crosschecked all remaining references. We extracted bibliographic and study specific data, information on the involvement methods, their context of application, reported outcomes and the characteristics of the participating people with dementia. Regarding the context of application, we classified information on the stages of policy and guideline development. For policymaking, we used a four‐step model of the policy process, consisting of: (i) agenda setting, (ii) policy formulation, (iii) implementation, and (iv) evaluation [20]. For guideline development, we used a simplified version of the guideline process, consisting of six steps: (i) defining the guideline scope, (ii) recruiting a working group, (iii) searching for evidence, (iv) developing best‐practice recommendations, (v) consulting interest‐holders, and (vi) disseminating and implementing the guideline [21].

We analysed each involvement method according to the public engagement typology proposed by Rowe and Frewer [15]. Herein three types of ‘public engagement’ (we use the term ‘involvement’) are distinguished, based on the information flow: public communication (information flows from the sponsor to the public representatives), public consultation (from public representatives to the sponsor), and public participation (in both directions) [15]. Additionally, the typology comprises six mechanisms associated with maximising the effectiveness of public engagement, that is, maximising the transfer of relevant information. The mechanisms relate to the (i) controlled versus uncontrolled participant selection, (ii) facilitated versus nonfacilitated information elicitation, (iii) open versus closed response mode, (iv) flexible versus set information input, (v) face‐to‐face (FTF) versus non‐FTF information transfer, and (vi) structured versus unstructured aggregation of participants' contributions [15].

Data S2 provides the final data extraction template, including information on the policy and guideline processes and the public engagement typology. For data analysis, we tabulated the extracted data using frequencies and percentages.

### 2.7 Interest‐Holder Consultation

We involved the German ‘Dementia and Research’ working group twice during this review. The working group is hosted by the German Alzheimer Association and consists of four people with dementia interested in dementia care research. The group meets monthly to discuss current dementia research projects. An employee of the Alzheimer Association who is familiar with the participants moderates the group meetings.

During the first online meeting in August 2023, we discussed the review protocol to assess whether participants were interested in supporting the interpretation of results. For the second online meeting in September 2024, we provided a short written summary of the results and the discussion points in advance, as participants had not received any additional information since the first meeting. We then presented the review findings and asked participants to provide their opinions on the involvement methods and the measures taken to facilitate information elicitation in these methods. The key messages from this interest‐holder consultation provided us with guidance and a solid basis for interpreting our findings and are presented in the discussion section.

## 3 Results

### 3.1 Characteristics of Included Reports

**FIGURE 1:**
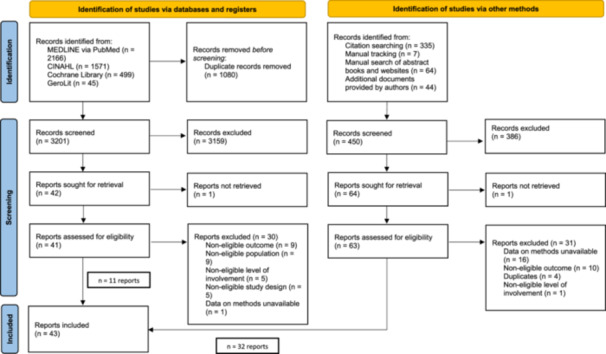
Flowchart of study selection and inclusion process.

Database searches yielded 3201 unique references. We assessed 41 references in full text, of which 11 met our inclusion criteria. Our additional search strategies yielded 450 references, of which we assessed 63 as full text and included 32. In total, we included 43 reports [22, 23, 24, 25, 26, 27, 28, 29, 30, 31, 32, 33, 34, 35, 36, 37, 38, 39, 40, 41, 42, 43, 44, 45, 46, 47, 48, 49, 50, 51, 52, 53, 54, 55, 56, 57, 58, 59, 60, 61, 62, 63, 64]. Figure 1 displays the selection process. During screening and data extraction, we sought additional information from 48 authors via e‐mail, and had online meetings with two authors. We were unable to get in touch with six authors.

We included original studies and research reports, policy documents, guideline documents and other reports, most of which were published by dementia or aged care organisations (details on the publication types are displayed in Data S3). Of the six included study reports, three used a quantitative design [22, 23, 34], two used mixed or multimethod designs [35, 38] and one was a case study [24]. We included one study protocol of a qualitative study [40].

All reports were published between 2009 and 2024 (median 2020), with more than 80% (*n* = 36) published in the last 10 years (2014–2024). Almost half (*n* = 18) were published in the United Kingdom, of which 10 were from Scotland. Overall, about two thirds (*n* = 28) were published in Europe, 10 reports in North America, three in Australia, one in China, and one report was a multinational collaboration.

### 3.2 Involvement Methods

Across all included reports, we extracted information on 35 methods used to involve people with dementia in policymaking or guideline development. These cover 22 unique methods, of which six methods were used in several instances (engagement events, focus groups, individual interviews, serving as members of working groups, surveys and online surveys). We categorised the methods into six groups based on similarity, which are shown in Table 1.

**TABLE 1:** Description of methods for the involvement of people with dementia.

Category name	Involvement method	Description	Examples of policies/guidelines
Focus groups and interviews	Individual interviews [53, 56, 59, 60]	Exploring views, experiences, or perceived needs for action from the point of view of people with dementia, either in individual semistructured interviews or in focus groups, hosted by a researcher, facilitator or moderator. Relevant information or the main questions may be provided in advance. Interviews may take place in people's homes or at the facilities of responsible organisations.	Dementia strategy (national) [56]
	Focus groups [29, 30, 31, 36, 53, 54, 56, 59, 60]		Dementia strategy (state) [29] Dementia strategy (national) [56] Social care guideline on critical wandering in dementia [36] National aged care design principles and guidelines [59, 60]
Surveys and questionnaires	(Online) Survey [23, 35, 59, 60, 62, 63, 64]	Rating importance, usability, comprehensibility, information presentation, satisfaction etc. of guideline or policy drafts or resources, guideline questions, or services. Surveys may include scales such as Likert‐Scales. Telephone support may be in position for people with dementia who are unable to fill out online surveys (e.g., [63, 64]).	Social care guideline on critical wandering in dementia [35] Legislation for the establishment of the national care service, focussing on social care in Scotland [62] Clinical practice guideline on the use of amyloid positron emission tomography (PET) imaging in patients with or at risk of dementia [23]
	Public consultation [48, 49, 51, 52]	Gathering feedback on topics of interest via open‐ended questions accessible to the public. Public consultations may be part of the national policy process (e.g., in Scotland).	Dementia strategy (national) [48, 49, 51, 52]
	Online questionnaire [41, 42]		National action plan to support recovery from Covid‐19 of people with dementia and their carers [41, 42] Dementia Strategy (national) [48]
	Public comment [24]	Providing open feedback on draft guidelines on a publicly accessible website, with the 30‐day comment period being announced to patient representatives, professionals, associations and the broader public.	Clinical practice guideline on the use of amyloid positron emission tomography (PET) imaging in patients with or at risk of dementia [24]
Public events	Engagement events [41, 42, 49]	Exploring experiences, needs or challenges in small groups at openly accessible events (online or in‐person), with other stakeholders and interested persons present and/or participating. Groups are not exclusive to people with dementia, and may be led by moderators or facilitators. Creative methods for information elicitation may be used (e.g., imagination workshops [62, 64]). Stakeholders from social care, research, technology, informal carers, aged care or dementia care associations etc. may be invited. Events may comprise several other activities forexample, presentations from researchers, policymakers, ministers etc [37].	National action plan to support recovery from Covid‐19 of people with dementia and their carers [41] Dementia strategy (national) [48]
	Key stakeholder forum [37]		Social care guideline on critical wandering in dementia [37]
	Dialogue meetings [58]		Dementia strategy (national) [58]
	Online engagement sessions with creative methods [62, 64]		Legislation for the establishment of the national care service, focussing on social care in Scotland [62]
	Discussions and creative workshops (conference) [62, 64]		Legislation for the establishment of the national care service, focussing on social care in Scotland [62]
Meetings with decision makers	Meeting with select committee members [43, 44, 45]	Discussing topics of interest and perspectives of people with dementia with decision‐makers such as government ministers and officials, select committee members, social security administration staff, Department of Health staff, or staff from associations responsible for the development of clinical practice guidelines. Discussions may be led by facilitators or decision makers. People with dementia may answer questions, give speeches, read prepared statements, or discuss topics important to them. People with dementia may receive information on topics or (dementia‐adapted) materials in advance, or may receive support in preparation of the meetings, for example, in preparing their statements and speeches.	Mental capacity act [43, 45]
	Meeting with government ministers or Prime Minister [53, 55]		Dementia strategy (national) [53, 55]
	Hearing with social security administration [57]		Legislation on the compassionate allowance [57]
	Listening session with medical association staff [57]		Clinical practice guideline [57]
	Roundtables with representatives from the Department of Health and the national dementia strategy [25, 27, 28]		Dementia strategy (national) [26]
Serving on working groups	Serving on guideline development/drafting groups [22, 24]	Involving people with dementia in activities of (multidisciplinary) working groups at one or several stages during the policy or guideline development process. People with dementia may be involved in developing guideline questions, sharing their experience with guideline/policy topics, drafting or refining materials, resources or recommendations, prioritising or specifying topics, overseeing development processes, implementation or delivery of strategies, providing cultural insight or advising jury members.	Clinical practice guideline on the use of amyloid positron emission tomography (PET) imaging in patients with or at risk of dementia [22, 24]
	Serving on working groups [46, 47, 55]		National dementia guidelines on disclosing and communicating a diagnosis of dementia [47]
	Serving on Research User Groups (RUGs) [39, 40]		Social care guidelines on recognition and management of hearing and vision impairment in people with dementia [39, 40]
	Serving as jury advisers [59, 60, 61]		National aged care design principles and guidelines [59, 61]
	Serving on dementia working groups (SDWG, National Dementia Lived Experience Panel) [50, 55]		Dementia strategy (national) [48, 55]
Multiple‐step methods	Interviews with subsequent confirmatory survey [34]	Exploring experiences and needs in semistructured individual interviews via telephone or web conference, conducted by a researcher. People with dementia may choose to participate in the presence of a family member. Relevant information is provided in advance. The subsequent survey intends to investigate whether the suggestions from the interviews were adequately implemented.	Social care guideline on critical wandering in dementia [34]
	Policy café [32, 33]	Exploring the perspectives of people with dementia on policy‐relevant issues in an informal group setting moderated by a facilitator, who prompts questions, helps record the discussion, and supports people with dementia if needed. After the discussion, people with dementia write their personal priorities on cue cards, sort them by similarity, and prioritise them to identify final key messages.	Legislation on home care [32]
	Delphi [38]	Exploring the perspectives and priorities of people with dementia via open‐ended questions from individual phone interviews conducted by researchers to generate statements. After analysis of results and statement generation (performed by researchers), people with dementia prioritise the statements' importance by rating them on a five‐point Likert scale. During this second stage, additional suggestions may be provided.	Integrated action plan for local health response across the primary and secondary care settings to dementia [38]

We found that methods traditionally used for qualitative research such as **focus groups and individual interviews** were employed to explore the needs and experiences of people with dementia for planned policies [29, 30, 56] or guidelines [59, 60], or to assess their opinions on guideline drafts [36]. Focus groups were either open for other participants such as informal carers or aged persons [56, 59], or organised exclusively for people with dementia [29, 30, 36]. Similarly, quantitative research methods such as **surveys and questionnaires** were used to rate the importance of guideline questions [23], to assess the usability and design of guideline drafts [35], or to gather interest‐holder input for planned guidelines [59, 60]. With the exception of one survey created specifically for people with dementia [62, 64], all other surveys were accessible to participants without dementia [23, 35, 59, 60]. In contrast to surveys, public consultations [48, 49, 52] and online questionnaires [41, 42] allowed open feedback, and were only used for policymaking purposes. One study reported a public comment during the development of a clinical practice guideline as part of the standard guideline methodology [24]. However, it remained unclear whether any people with dementia participated, as some commenters remained anonymous [24]. The report still met our inclusion criteria as it described the public comment as a method aiming to involve people with dementia in guideline development, regardless of the number of participants with dementia.

People with dementia participated in different **public events** to discuss policy or guideline‐relevant topics with potential users, people with lived experience, informal carers, and other interest‐holders. However, the differences between these methods remain partially unknown due to incomplete reporting. We identified several events where people with dementia voiced their opinions regarding future dementia care policy [41, 42] or the development of dementia strategies in Scotland [48, 52] and Norway [58]. One method consisted of a creative conference workshop in which the participants with dementia were encouraged to ‘build’ an ideal National Care Service by designing posters or using imagination exercises [62, 64]. Most public events were organised for health or social policy purposes. Additionally, we identified a ‘key stakeholder forum’ with carers, technology industry representatives, researchers and people with dementia, organised during the development of a social care guideline [37]. However, it remained unclear in which of the reported activities (panel discussions, round table discussions) people with dementia participated.

**Meetings with decision‐makers** were mostly used in health and social policy contexts. We found reports of people with dementia meeting with Scottish government ministers [53, 55, 62, 64] and a UK House of Lords select committee [43, 44, 45]. Other meetings took place with the Irish Department of Health staff and representatives from the Irish National Dementia Strategy [25, 26, 27, 28], and staff from the US Social Security Administration (SSA) [57]. One medical association organised a listening session of association staff with people with dementia during the development of a clinical practice guideline [57].

The involvement of people with dementia by **serving on working groups** mainly occurred in the context of guideline development. Working groups required continuous commitment instead of one‐time involvement. We found reports of people with dementia supporting question development for a clinical practice guideline [22, 24], overseeing guideline development in steering committees, supporting development of recommendations in topic guideline creation panels, and providing cultural insight via community working groups [46, 47]. Members of the Scottish Dementia Working Group (SDWG) supported the development of the Scottish National Dementia Strategy by serving on working groups, although their responsibilities were not described in detail [55]. Finally, people with dementia served as advisers to an expert jury for the Australian guidelines on aged care design [59, 61].

Lastly, we formed one category of **multiple‐step methods**. One study reported the use of semistructured individual interviews with a subsequent confirmatory survey as a way of refining a social care guideline [34]. Another study used a Delphi approach to create initial statements on dementia care needs, which participants subsequently rated according to importance [38]. One report described a policy café, which employed the world café method to discuss policy‐relevant issues in a facilitated group setting [32]. Following the discussion, participants identified key messages by sorting and prioritising cue cards with their personal priorities [32].

### 3.3 Theoretical Underpinnings

No theoretical underpinning was reported in a majority of 24 methods. In six methods, PPI was referred to as a general principle [22, 23, 24, 32, 39]. Two methods reported patient [39] or user‐centredness [37] as underpinnings, and two methods referred to participatory research [29, 30] or participatory action research [46, 47]. Additionally, general principles of coproduction [32, 33], PANEL principles [50], or the Delphi approach [38] were referred to by one method each. While the involvement of people with dementia in the coproduction of a policy café was reported comprehensibly (e.g., identifying discussion topics for the event) [32, 33], it remained unclear which role the PANEL principles played for the work of the National Dementia Lived Experience Panel during the development of the Scottish National Dementia Strategy [50].

### 3.4 Barriers and Facilitators

For most involvement methods, neither barriers nor facilitators were reported. We found some information on general PPI barriers, such as unsuitable group sizes for working groups or medical jargon [22, 24]. Barriers to the involvement of people with dementia in particular were reported twice only, but information on how it was attempted to overcome these barriers was not provided. Similarly, some methods reported on general PPI facilitators relating to the establishment of team principles [47] or the impact of adequate group facilitation [47]. Reports on two methods specified facilitators to the involvement of people with dementia, emphasising the use of creativity [32] and a variety of contribution methods [47]. Table 2 displays all the reported barriers and facilitators.

**TABLE 2:** Reported barriers and facilitators (B&F).

	Barriers (methods)	Facilitators (methods)
General B&F reported for the involvement methods	Medical jargon (guideline development groups) [22] Patient representatives (not all had dementia) raised topics not clearly linked to the guideline (guideline development groups) [22] Patient representatives (not all had dementia) consistently rated survey items as ‘extremely important’ (survey) [23] Group size of 16 participants was too large to work together (guideline drafting group) [24]	Offering a hospitable environment (café tables, flowers, refreshments) (Policy Café) [32] Guideline development groups: Having participants' expenses paid for [22] Participants having basic knowledge of medical terminology [22] Serving on working groups for a social care guideline: Setting team principles to create a safe space for everyone [47] Having strong facilitators who make sure quieter individuals are not passed over [47] Having facilitators from the same ethnocultural background as the participants [47] Giving an introduction to person‐centred language [47]
B&F reported specifically for participants with dementia	Recruiting people with dementia took a long time (serving on working groups for a social care guideline) [47] Balancing in‐depth discussion with the available time (max. 2,5 h according to people with dementia) (Policy Café) [32]	Including creative methods, for example, drawing, illustrations, different media (Policy Café) [32] Providing alternative ways to contribute (chat function, e‐mails, calls) (serving on working groups for a social care guideline) [47]

### 3.5 Policies and Guidelines

Of the 35 involvement methods we identified, 21 (60%) were employed in the context of policymaking. Most of these methods (*n* = 11) were applied during the development of national [25, 48, 52, 53, 55, 56, 58] or state [29, 30] dementia strategies, or health and social care legislation [32, 45, 57, 62, 63, 64]. In five instances, several involvement methods were used for the same policy issue [42, 49, 55, 56, 64]. We found evidence of the involvement of people with dementia at all policy stages, with the vast majority of methods applied during the first (agenda setting, *n* = 18) or second stage (policy formulation, *n* = 6) of the policy process.

We found reports on the use of 14 involvement methods (40%) for the development of health or social care guidelines. Clinical practice guidelines addressed the use of imaging techniques in the diagnosis of dementia (*n* = 4) [22, 23, 24], or the prescription of antipsychotics in dementia‐related agitation and psychosis (*n* = 1) [57]. Social care guidelines addressed critical wandering (*n* = 4) [34, 35, 36, 37], hearing and vision impairment in people with dementia (*n* = 1) [39], communication of the dementia diagnosis (*n* = 1) [46, 47], or design principles for residential aged care accommodation (*n* = 3) [59, 60, 61]. In three instances, several methods were used for the same guideline [24, 34, 60]. Most involvement of people with dementia took place during the first stage (identification of the guideline scope, *n* = 7) or second stage (engaging with key interest‐holders and recruiting working groups, *n* = 7) of the guideline process. Additionally, eight methods were used during the fifth stage, that is, the interest‐holder consultation stage.

### 3.6 Outcomes

Assessing the involvement outcomes was challenging as the reporting was often incomplete. For 11 methods, contributions of people with dementia were used for clearly described subsequent stages [22, 23, 24, 32, 34, 35, 36, 37, 39, 47]. For instance, we found several publications on the development of a social care guideline on critical wandering in dementia, involving people with dementia at several stages. Starting with the decision that a guideline should be developed, which was made at a key stakeholder forum together with people with dementia [37], the development, iterative refinement based on interest‐holders' feedback, and dissemination are described [34, 35, 36]. However, for 16 methods we were unable to assess how the contribution of people with dementia was incorporated into the final guideline or policy [25, 26, 27, 28, 29, 30, 31, 41, 42, 43, 45, 48, 49, 50, 51, 52, 53, 54, 56, 57, 58, 59, 60, 61]. One method reports a policy decision made following the involvement of people with dementia [57]. After a hearing with the US Social Security Administration (SSA), in which two people with early‐stage dementia reported their personal dementia experiences to SSA staff, young‐onset dementia became eligible for the compassionate allowance. However, it is unclear to what extent this hearing actually influenced the policy decision [57].

### 3.7 Participants

The reporting of participants' characteristics was generally scarce. We did not extract all of the following information from the reports, but received additional information from the authors. The number of participants with dementia ranged between one and 45, with 12 methods reporting no precise number [41, 42, 49, 52, 55, 57, 58, 60, 62, 64]. Participants' age ranged from 46 to 79 years, but was only reported for six methods [35, 36, 38, 39, 56, 57]. Dementia subtypes were reported for four methods [32, 39, 47, 57], with all participants having Alzheimer's or mixed dementia. Dementia stages were reported for 11 methods, with the majority of participants described as having mild cognitive impairment (MCI) [22, 24, 38], mild to moderate or early to mid‐stage dementia [34, 36, 47, 56, 57]. Only one study reported participants with moderate dementia, though they made up only around 7% of the sample [38]. Participants' sex was reported for seven methods [30, 32, 35, 38, 56, 57, 61], with a total of 50 women and 31 men participating.

We found eight involvement methods that were conducted exclusively with people with dementia [32, 36, 38, 45, 56, 61, 64], while for three methods, reports did not specify whether other people participated [49, 55, 64]. The remaining 24 methods involved other participants, most frequently informal carers and family members, healthcare professionals, researchers, physicians or health and social care organisation staff. While the majority of reports did not specify the target group of the involvement methods (*n* = 23), seven methods specified targeting people with mild or early‐stage dementia [34, 57, 62, 63, 64] and one method targeted people with dementia with either hearing or vision impairment [39]. The recruitment of participants with dementia occurred most frequently via pre‐existing working groups or self‐help groups [25, 26, 29, 30, 32, 33, 41, 42, 43, 44, 45, 48, 51, 52, 53, 55, 57, 62, 64], dementia or aged care organisations [22, 23, 24, 35, 37, 53, 54, 56, 59, 61], or previous contacts and key informers [34, 48, 49, 51, 52, 62, 64]. Information on the participants of each method and the recruitment strategies are displayed in Data S4.

### 3.8 Involvement Mechanisms

No involvement methods represented public communication, indicating information flow from the PPI sponsors to the public representatives. Of the 35 involvement methods we identified, 28 represented public consultation, and seven constituted public participation. All public participation occurred either when people with dementia served as members of working groups (*n* = 6) [22, 24, 46, 47, 49, 55, 61] or during public events (*n* = 1) [37]. Although many reports did not provide much detail, we analysed the underlying mechanisms of the involvement methods according to the public engagement typology [15]. Here, we focus on the mechanisms least recognisable by the name of the involvement method alone. Data S5 displays the detailed analysis of each involvement mechanism for all the included involvement methods.

We found that 26 methods controlled the selection of participants, that is, only the target group could participate. However, of these only eight addressed exclusively people with dementia [32, 36, 38, 45, 56, 61, 62, 64]. Seven methods had uncontrolled participant selection, for example, openly accessible dialogue meetings [58], a public comment [24] or surveys [23, 60, 64], meaning anyone could participate. Although these methods were not designed to involve exclusively people with dementia, uncontrolled participant selection tended to result in only a few participants with dementia. For instance, an online public consultation received 167 responses, but only seven respondents had dementia [49]. The details on the numbers of participants with dementia are displayed in Data S4.

Of the 35 identified methods, over two thirds (*n* = 27) facilitated the elicitation of information from participants, most frequently through interviewers, moderators or discussion facilitators. While many involvement methods have facilitation measures built into the standard procedures (e.g., an interviewer facilitates an interview), some employed additional assistance specifically aimed at people with dementia, such as providing relevant materials or questions in advance [34, 45, 61]. For the development of a clinical practice guideline, people with dementia received a dementia‐adapted two‐page version of the guideline beforehand [57]. We also found one facilitated method where people with dementia were asked to record their experiences with a web‐based guideline in a diary for 3 weeks before discussing these experiences in a moderated focus group [36]. Members of dementia working groups received assistance from staff who supported them in preparing their statements for hearings, or prepared them for discussion topics [55, 57, 64]. For surveys [23, 35], public comment or consultation [24, 49], no facilitation was reported.

Finally, the public engagement typology lists structured aggregation of participant information as an involvement mechanism. This mechanism assesses whether all individual contributions are systematically and equally aggregated into a central statement or message as a result of the involvement [15]. We found nine methods using structured aggregation, with three methods aggregating survey data [23, 35, 64] and three methods reaching group consensus, either by rating priorities in a Delphi approach [38], via cue cards [32], or in working groups [47]. For the development of guideline questions, a moderator facilitated aggregation of all contributions by supporting the groups in formulating questions according to the PICOT format [22, 24]. Seventeen involvement methods did not employ structured aggregation, most of which constituted group meetings or discussions, either with or without decision‐makers.

## 4 Discussion

Our review provides the first comprehensive overview of the involvement of people with dementia in health or social policy and guideline development. We identified 35 involvement methods and organised them in six categories, based on similarity. Our findings indicate that people with dementia are most frequently involved at the macro‐level of care by means of consultations during early stages of policy or guideline development. The small number of included study reports suggests that the involvement of people with dementia is currently not a subject of extensive scientific enquiry. Most of the included reports were published after 2013, indicating an increasing importance of the topic; however, our hand search was also limited to the past 10 years. The reporting of the involvement methods and participant characteristics was often incomplete, making it difficult to assess the outcomes of involvement or the barriers and facilitators.

Our findings suggest a limited use of target group‐specific or dementia‐adapted involvement methods. We found that a considerable amount of involvement took place via methods typically used for research purposes, for example, interviews, focus groups, questionnaires or surveys. While some involvement methods were adapted to accommodate participants with dementia specifically (e.g., providing questioning routes [34] or guideline drafts [57] in advance), most methods were not, or the adaptations were not reported. Furthermore, all involvement methods required substantial levels of verbal skills to express one's opinions orally or in writing, respond to questions, or react to what others said. This is consistent with previous findings on PPI for people with dementia in research [9]; however, it raises the question whether relying on verbal communication alone is a suitable approach, given the potential impact of dementia on the ability to communicate verbally [65]. While medical jargon was reported as an involvement barrier [22], general language comprehension was not reported as being a negative impact on the involvement. For all involvement methods relying on verbal communication, the available recommendations on the appropriate use of language for communication with people with dementia should be taken into account [66].

Previous studies have used novel data collection techniques to involve people with dementia in research (e.g., photo elicitation, storytelling exercises, capturing nonverbal communication etc.) [4], or offered flexibility regarding the data collection methods to accommodate people with dementia [4]. A recent review on the involvement of older adults in health policymaking suggests that the use of creative or multiple methods (e.g., gallery walks, art sessions, user panel workshops etc.) may lead to more effective involvement and a more meaningful impact compared to traditional involvement approaches [67]. While we found several PPI initiatives employing multiple involvement methods for the same policy or guideline, we found only two methods declaring creative or innovative data collection approaches, that is, a conference workshop using an imagination technique and a policy café [32, 62, 64]. The reason for these differences in use of involvement methods remains unclear as the majority of reports we included contained neither theoretical underpinnings nor clear involvement rationales. We also found no evidence of involvement via methods previously used in policy contexts for people without dementia (e.g., citizen juries [68] or deliberative democracy sessions [69]).

Based on our analysis using the public engagement typology [15], most involvement constituted public consultation, indicating a unidirectional flow of information from people with dementia to the PPI sponsors. Public participation, which is characterised by a bidirectional flow of information, was reported less frequently, but almost exclusively when people with dementia served on working groups for extended time periods. In our interest‐holder consultation with a dementia working group, the participants pointed out that they considered continuous involvement more likely to lead to an increase in knowledge, which was desirable, as participants themselves should also benefit from their involvement. However, our findings suggest that most working groups involving people with dementia included other participants, such as physicians, guideline experts [22, 24] or healthcare professionals [46]. Our interest‐holders emphasised that they considered heterogeneous groups with members without dementia difficult to navigate. They pointed out that involving one single person with dementia in a working group with many other participants, which we found in several instances [22, 24, 46, 47], was an unsuitable approach. An individual participant with dementia would likely feel left alone and not have the courage to speak out. While our interest‐holders considered working groups as the most suitable involvement approach overall, they suggested that dementia‐specific working groups without other participants, but with considerate moderation and facilitation were the most likely to encourage involvement.

We acknowledge that the opinions of our interest‐holders may be biased and favour working groups, as they were members of a working group themselves, and stated that they were less familiar with the other involvement methods. However, our results suggest that the involvement in working groups accounted for the majority of public participation, and bidirectional flow of information corresponds more closely with some PPI principles (responding to PPI contributions, providing updates to contributors [1]) than the unidirectional flow of information found in public consultation. Additionally, the continuous involvement we found to be a distinguishing feature of working groups may better enable the implementation of other PPI principles (commitment to ongoing collaboration, re‐evaluating and tailoring involvement approaches [1]) compared to the mostly one‐time consultations we found. The demand for continuity of involvement is also in line with the WHO recommendations for meaningful engagement, which emphasise the need for iterative and continuous involvement over one‐time involvement, which is often experienced as disrespectful and undermining [70]. We therefore consider the points raised by our interest‐holders to be pertinent and emphasise that they have clearly spoken out in favour of continuous involvement in working groups as a PPI method.

Only a few included reports listed involvement barriers and facilitators, reported on how the barriers were dealt with, or reported evaluation results of the involvement methods. This makes it difficult to use the experiences gained for future PPI initiatives. Especially the lack of reported involvement barriers seems remarkable, as the involvement of people with dementia is generally described as challenging [5, 6]. Similarly, many reports mentioned that the involvement outcomes were utilised for the intended purposes, but few outlined how the contributions from people with dementia affected the final policies or guidelines. This is in line with findings of the few previous studies evaluating PPI at the macro‐level of care, which suggest that involvement of patient representatives does not equate to policy impact [71, 72]. We attribute the lack of information on involvement outcomes partly to the included publication types and the large portion of nonscientific reports. While guidance for the comprehensive reporting of PPI is available [73], it may not be well‐established in nonacademic areas; consequently, only one of the included reports referred to this guidance [40]. However, the incomplete reporting reveals a broader and more serious issue as it questions some of the fundamental PPI principles such as transparency, shared knowledge, and shared power [1]. Unclear reporting on the involvement impact and failure to recognise the contributions of patient representatives, however well‐intentioned, suggests tokenism. Researchers need to consider and address power inequities when designing PPI initiatives, and ensure that contributions are included in decision‐making [70]. We therefore call on researchers and policymakers to report transparently on how people with dementia were involved, what they contributed, how their contributions were integrated into decision‐making processes, and which impact their involvement had on the overall outcomes of the PPI initiative.

### 4.1 Strengths and Limitations

Our review has limitations. As indicated by previous studies [32], we did not expect to find many original study reports on the involvement of people with dementia at the macro‐level of care. We therefore attempted to capture the breadth of publications by combining database searches with additional search strategies. However, we were only able to consider grey literature in English and German. This may have resulted in relevant PPI initiatives being overlooked. For database searches, we limited our search to health and medical databases, although health and social care policymaking may also be found in policy‐specific publications not indexed in these databases. Many involvement methods were employed during the development of dementia strategies and plans; however, we did not conduct hand searches for all the published dementia strategies, as this was beyond the scope of our work. For an overview on the involvement of people with dementia in dementia strategies only, we refer to two reports we found during our screening process, but did not include in the review as they lacked pertinent descriptions of involvement methods [74, 75]. Finally, we acknowledge that our analysis of the involvement mechanisms based on the public engagement typology [15] has some shortcomings. While we were able to apply the typology in most cases where people with dementia were involved during one‐time activities, the typology is not well suited to analyse ongoing involvement (e.g., in working groups), or multiple‐step involvement. Lastly, we would like to point out that structured aggregation of individual contributions as an involvement mechanism is based on the principle of equal representation in voting, assigning equal weight to each contribution (‘one person, one vote‘). The application of this principle seems reasonable given that the voices of people with dementia are often assigned little credibility in policymaking [76]. However, in many of the involvement initiatives we found, people with dementia were clearly outnumbered by participants without dementia. Our review did not investigate how these imbalances could be dealt with.

Still, we believe that analysing the involvement mechanisms poses a strength of this study. Other frameworks for PPI at the macro‐level of care focus on the power dynamics between researchers and PPI representatives, and categorise involvement according to the PPI contributors' degree of decision‐making authority (e.g., [13, 77, 78, 79]). We considered the public engagement typology [15] more suitable for this study as it focuses on maximizing the effectiveness of involvement, which may be particularly important for PPI with people with dementia in view of the challenges concerning recruitment and communication [65, 80]. We believe that presenting our results to a working group of people with dementia to discuss their implications has strengthened this study. Our search approach with extensive hand searches proved to be justified, as we identified the majority of relevant reports via additional search strategies, not via databases. Finally, we are confident that contacting the authors to provide additional, previously unpublished information equally strengthened the results and the relevance of our work.

## 5 Conclusion

Our scoping review shows that people with dementia are involved to a limited extent in the development of policies and guidelines. While different methods were used for involvement, our results suggest that only a few of these methods were dementia‐adapted. There is little evidence to date on the involvement of people with advanced dementia.

Based on our findings and interest‐holder consultation, we offer five recommendations for researchers or policymakers seeking to involve people with dementia at the macro‐level of care: (1) Choose involvement methods maximising the information transfer, or tailor methods to maximise information transfer. This will ensure that people with dementia can communicate and be involved effectively. Our analysis of the involvement methods and mechanisms may serve as a guidance. (2) Provide support and facilitation aimed specifically at people with dementia and tailored to individual needs to ensure that participants have the opportunity to contribute in a meaningful way. This may be achieved through trained facilitators, by providing relevant information and materials in advance, or by using assistive tools that enhance involvement. People with dementia face different challenges in terms of their involvement than people without dementia; this should be taken into account when designing the involvement initiatives. (3) Take measures to ensure that people with dementia are adequately represented in the sample. Relying on convenience sampling may result in either very few participants with dementia, or exclude those with varying levels of cognitive impairment. (4) Aim for ongoing involvement instead of one‐time consultation. Continuity may enable participants to become more deeply involved and benefit from involvement. Based on our interest‐holder consultation, we suggest involving people with dementia in continuously moderated, dementia‐specific working groups, and providing follow‐up and structured opportunities for discussion. (5) Document, report and evaluate all involvement methods, outcomes and impacts transparently. This is crucial for reproducibility and will enable further research into target‐group specific involvement methods and adaptations for people with dementia.

## Author Contributions

**Felix Bühler:** conceptualization, data curation, formal analysis, investigation, methodology, project administration, validation, visualization, writing – original draft, writing – review and editing. **Jennifer Geyer:** conceptualization, data curation, formal analysis, investigation, methodology, project administration, validation, visualization, writing – review and editing. **Gabriele Meyer:** conceptualization, funding acquisition, methodology, supervision, validation, visualization, writing – review and editing. **Anja Bieber:** conceptualization, funding acquisition, methodology, supervision, validation, visualization, writing – review and editing.

## Ethics Statement

The authors have nothing to report.

## Conflicts of Interest

The authors declare no conflicts of interest.

## Supporting information

Supplement 1: Database‐specific search strategies.

Supplement 2: Data extraction template.

Supplement 3: Characteristics of included reports.

Supplement 4: Characteristics of participating people with dementia.

Supplement 5: Analysis of involvement mechanisms according to the public engagement typology by Rowe and Frewer^1^.

## Data Availability

All data generated or analysed for this review are included in the article or its supplementary materials.
