# Supplementary material for: Methods for Involving People With Dementia in Health Policy and Guideline Development: A Scoping Review
Source: Health Expect. 2025 Apr 3;28(2):e70250. doi: 10.1111/hex.70250 (PMC11968782; doi:10.1111/hex.70250)
Supplement: Supplementary file 1 — Supplement 1: Database‐specific search strategies. [file HEX-28-e70250-s005.docx]

Supplement 1: Database-specific search strategies

| **Database** | **Date of Search** | **Search strategy** | **Hits** |
| --- | --- | --- | --- |
| Medline via PubMed | November 28, 2023 | (dementia[mesh] OR alzheimer*[tiab] OR dement*[tiab] OR mild cognitive impairment[tiab] OR mci[tiab])  AND  (community participation[mesh] OR empowerment[mesh] OR stakeholder participation[mesh:noexp] OR participat*[tiab] OR engag*[tiab] OR empower*[tiab] OR involv*[tiab])  AND  (advis*[tiab] OR advoca*[tiab] OR produc*[tiab] OR creat*[tiab] OR develop*[tiab] OR design*[tiab] OR codesign*[tiab] OR collaborat*[tiab] OR priorit*[tiab])  AND  (health policy[mesh] OR quality assurance, health care[mesh:noexp] OR guidelines as topic[mesh] OR policy making[mesh] OR health planning[mesh] OR legislation, medical[mesh] OR legislation, nursing [mesh] OR technology assessment, biomedical[mesh] OR social support[mesh] OR social welfare[mesh] OR polic*[tiab] OR reform*[tiab] OR guideline*[tiab] OR health system[tiab] OR health plan[tiab] OR health planning[tiab] OR legislat*[tiab] OR dementia strategy[tiab] OR “health technology assessment”[tiab] OR social care[tiab] OR social support[tiab] OR social service[tiab] OR social services[tiab] OR community service[tiab] OR welfare[tiab]) | 2166 |
| CINAHL via EBSCOhost | November 28, 2023 | ((MH dementia+) OR (TI alzheimer* OR AB alzheimer*) OR (TI dement* OR AB dement*) OR (TI "mild cognitive impairment" OR AB "mild cognitive impairment") OR (TI mci OR AB mci))  AND  ((MH "consumer participation") OR (MH empowerment) OR (MH "stakeholder participation") OR (TI participat* OR AB participat*) OR (TI engag* OR AB engag*) OR (TI empower* OR AB empower*) OR (TI involv* OR AB involv*))  AND  ((TI advis* OR AB advis*) OR (TI advoca* OR AB advoca*) OR (TI produc* OR AB produc*) OR (TI creat* OR AB creat*) OR (TI develop* OR AB develop*) OR (TI design* OR AB design*) OR (TI codesign* OR AB codesign*) OR (TI collaborat* OR AB collaborat*) OR (TI priorit* OR AB priorit*))  AND  ((MH "health policy+") OR (MH "quality assurance+") OR (MH "practice guidelines") OR (MH "policy making") OR (MH "Health and Welfare Planning+") OR (MH "legislation, medical+") OR (MH "legislation, nursing+") OR (MH "health impact assessment") OR (MH "social welfare") OR (TI polic* OR AB polic*) OR (TI reform* OR AB reform*) OR (TI guideline* OR AB guideline*) OR (TI "health system" OR AB "health system") OR (TI "health plan" OR AB "health plan") OR (TI "health planning" OR AB "health planning") OR (TI legislat* OR AB legislat*) OR (TI "dementia strategy" OR AB "dementia strategy") OR (TI "health technology assessment" OR AB "health technology assessment") OR (TI "social care" OR AB "social care") OR (TI "social support" OR AB "social support") OR (TI "social service" OR AB "social service") OR (TI "social services" OR AB "social services") OR (TI "community service" OR AB "community service") OR (TI welfare OR AB welfare)) | 1571 |
| Cochrane Library | November 28, 2023 | ([mh dementia] OR alzheimer*:ti,ab OR dement*:ti,ab OR "mild cognitive impairment":ti,ab OR mci:ti,ab)  AND  ([mh "community participation"] OR [mh empowerment] OR [mh ^"stakeholder participation"] OR participat*:ti,ab OR engag*:ti,ab OR empower*:ti,ab OR involv*:ti,ab)  AND  (advis*:ti,ab OR advoca*:ti,ab OR produc*:ti,ab OR creat*:ti,ab OR develop*:ti,ab OR design*:ti,ab OR codesign*:ti,ab OR collaborat*:ti,ab OR priorit*:ti,ab)  AND  ([mh "health policy"] OR [mh ^"quality assurance, health care"] OR [mh "guidelines as topic"] OR [mh "policy making"] OR [mh "health planning"] OR [mh "legislation, medical"] OR [mh "legislation, nursing"] OR [mh "technology assessment, biomedical"] OR [mh "social support"] OR [mh "social welfare"] OR polic*:ti,ab OR reform*:ti,ab OR guideline*:ti,ab OR "health system":ti,ab OR "health plan":ti,ab OR "health planning":ti,ab OR legislat*:ti,ab OR "dementia strategy":ti,ab OR "health technology assessment":ti,ab OR "social care":ti,ab OR "social support":ti,ab OR "social service":ti,ab OR "social services":ti,ab OR "community service":ti,ab OR welfare:ti,ab) | 499 |
| GeroLit | November 29, 2023 | (sww demenz ODER sww „kognitive beeinträchtigung“ ODER tit dement* ODER tit alzheimer*)  UND  (sww bürgerbeteiligung ODER sww empowerment ODER sww mitwirkung ODER sww mitbestimmung ODER sww bürgerschaftliches engagement ODER sww engagement ODER sww politische partizipation ODER sww politisches verhalten ODER sww teilnahme ODER tit partizip* ODER tit teilhabe ODER tit engag* ODER tit empower* ODER tit beteilig* ODER tit mitwirk* ODER tit mitbestimm*)  UND  (sww gesundheitspolitik ODER sww gesetz ODER sww gesundheitssystem ODER sww gesundheitswesen ODER sww qualitätssicherung ODER sww richtlinie ODER sww sozialrecht ODER sww altenhilfepolitik ODER sww altenpolitik ODER sww sozialplanung ODER sww altenhilfeplanung ODER sww sozialer dienst ODER sww soziale unterstützung ODER sww sozialpolitik ODER sww sozialreform ODER sww gesundheitliche versorgung ODER sww geriatrische versorgung ODER sww gerontopsychiatrische versorgung ODER sww planungsempfehlung ODER tit politi* ODER tit gesundheitspoliti* ODER tit sozialpoliti* ODER tit reform* ODER tit leitlinie* ODER tit richtlinie* ODER tit gesundheitssystem* ODER tit gesundheitsplan* ODER tit legislat* ODER tit gesetzgeb* ODER tit demenzstrateg* ODER tit gesundheitsdienst* ODER tit “soziale unterstützung” ODER tit “soziale dienste” ODER tit hilfe* ODER tit wohlfahrt*) | 45 |
