# Supplementary material for: Methods for Involving People With Dementia in Health Policy and Guideline Development: A Scoping Review
Source: Health Expect. 2025 Apr 3;28(2):e70250. doi: 10.1111/hex.70250 (PMC11968782; doi:10.1111/hex.70250)
Supplement: Supplementary file 2 — Supplement 2: Data extraction template. [file HEX-28-e70250-s003.docx]

Supplement 2: Data extraction template

| **Part 1: Characteristics of included references** | | | |
| --- | --- | --- | --- |
| Authors |  | | |
| Title |  | | |
| Year of publication |  | | |
| Country of origin |  | | |
| Publication type (research paper, report, editorial, study protocol, book (chapter), conference abstract, other [specify]) |  | | |
| **ONLY** for empirical studies:  Study design (RCT, case study/series, review etc.) |  | | |
| **ONLY** for empirical studies:  Stated objective(s) |  | | |
| **ONLY** for empirical studies:  Key findings regarding the objective(s) (*short* summary) |  | | |
| **Part 2: Methods of involvement in health policy and guideline development** | | | |
| Name of involvement method(s) |  | | |
| *Short* description of involvement method(s) |  | | |
| Reported theoretical underpinnings of the involvement method(s) |  | | |
| Reported target groups of the involvement method(s) |  | | |
| Type of policy document or guideline under investigation |  | | |
| Stage(s) of development in which people with dementia were involved *(4 stages for policies and National Dementia Plans, 6 stages for guidelines,* *details* *see below*) |  | | |
| Reported outcomes of the involvement of people with dementia (e.g. input into policy formulation, taken decisions, what happened with the results in the policy context?) |  | | |
| Reported barriers and facilitators for the involvement method(s) | **Barriers** | | **Facilitators** |
| Mechanism class (according to typology by Rowe & Frewer, *details see below*) |  | | |
| **Part 3: Participants** | | | |
| Reported characteristics of participating people with dementia | Number |  | |
|  | Age (range) |  | |
|  | Sex |  | |
|  | Dementia subtypes by frequency (as reported) |  | |
|  | Dementia stages by frequency (as reported) |  | |
| Recruitment of participating people with dementia (*short* *description*, project based? pre-existing working group? etc.) |  | | |

**Additional information 1: stages of development for policies and guidelines**

| **Stages of the policy process** | **Stages of guidelines development** |
| --- | --- |
| Wegrich, K., & Jann, W. (2007). Theories of the Policy Cycle. In F. Fischer, G. Miller, & M. S. Sidney (Eds.), *Public Administration and Public Policy: Vol. 125. Handbook of Public Policy Analysis: Theory, Politics, and Methods* (pp. 43–62). Taylor & Francis. <https://doi.org/10.1201/9781420017007.pt2>   1. **Agenda setting: problem recognition and issue selection**  - Recognising a (social/care) problem or selecting a specific issue for future policymaking among other issues - Considering or prioritising different potential issues for future policymaking - Moving a recognized issue to the political agenda      1. **Policy formulation and decision-making**  - Transforming expressed problems, proposals and demands into (government) programs - Defining objectives (what should be achieved with the policy?) - Considering or prioritising different action alternatives - Considering required and available resources for these action alternatives - Resolving conflicting interests between different stakeholder groups  1. **Implementation**  - Executing or enforcing a policy - Activities to achieve the goals of the policy - Specifying program details (who does what when and how?) - Allocating resources (budgets, which personnel will execute the program?)  1. **Evaluation**  - Appraising the impacts of the policy against the intended objectives - Investigating unintended consequences of policies - May take place at different stages throughout the policy cycle, not only in the end! | De Leo, A., Bloxsome, D., & Bayes, S. (2023). Approaches to clinical guideline development in healthcare: A scoping review and document analysis. *BMC Health Services Research, 23*(1), 37. <https://doi.org/10.1186/s12913-022-08975-3>   1. Identifying the need for and scope of the guidline 2. Recruitment of an interdisciplinary working group and engaging with key stakeholders 3. Searching for evidence 4. Developing best practice recommendations 5. External review and stakeholder consultation 6. Dissemination and implementation of recommendations |

**Additional information 2: Variables of public engagement**

| **From:** Rowe, G., & Frewer, L. J. (2005). A Typology of Public Engagement Mechanisms. *Science, Technology, & Human Values, 30*(2), 251–290. <https://doi.org/10.1177/0162243904271724>  **Three classes for public engagement, differentiated according to the flow of information**   \| **Flow of information** \| \| \| \| --- \| --- \| --- \| \| Public communication \| \| \| \| Sponsor \| **→** \| Public representatives \| \| Public Consultation \| \| \| \| Sponsor \| **←** \| Public representatives \| \| Public Participation \| \| \| \| Sponsor \| **↔** \| Public representatives \| | **Six mechanism variables**   1. Participant selection    - Is there control over the participant public representatives?    - Levels: controlled/uncontrolled 2. Facilitation of information elicitation    - Is facilitation in place to support the information elicitation process (e.g. an interviewer in focus groups, facilitator in citizen juries other methods of elicitation)    - Levels: yes/no 3. Response mode    - Can respondents choose among several response options (closed) or give free responses (open)?    - Levels: open/closed 4. Information input    - Do public representatives have control over the information provided within the public communication?    - Levels: set information/flexible information 5. Medium of information transfer    - Is information transferred face-to-face (FTF) or via other, non-face-to-face transfer methods?    - Levels: FTF/Non-FTF 6. Facilitation of information aggregation    - Is facilitation in place to merge participants’ responses or opinions into a composite response accurately combining all relevant information?    - Levels: structured combination/unstructured combination |
| --- | --- | --- | --- | --- | --- | --- | --- | --- | --- | --- | --- | --- | --- | --- | --- | --- | --- | --- | --- | --- | --- | --- |
