# Supplementary material for: Methods for Involving People With Dementia in Health Policy and Guideline Development: A Scoping Review
Source: Health Expect. 2025 Apr 3;28(2):e70250. doi: 10.1111/hex.70250 (PMC11968782; doi:10.1111/hex.70250)
Supplement: Supplementary file 3 — Supplement 3: Characteristics of included reports. [file HEX-28-e70250-s004.docx]

Supplement 3: Characteristics of included reports

| **Reference** | **Year** | **Country** | **Publication Type** | **Study Design** | **Number of methods described** |
| --- | --- | --- | --- | --- | --- |
| Armstrong et al.^1^ | 2018 | USA | Original study report | Parallel Group Study | n = 1 Guideline development groups |
| Armstrong et al.^2^ | 2019 | USA | Original study report | Survey | n = 1 Survey |
| Armstrong et al.^3^ | 2020 | USA | Original study report | Case Study | n = 2 Public comment Guideline drafting group |
| Begley^4^ | 2014 | Ireland | Conference abstract | n.a. | n = 1 Roundtables |
| Ireland Department of Health^5^ | 2014 | Ireland | Policy document | n.a. |  |
| Alzheimer Society of Ireland^6^ | 2013 | Ireland | Report | n.a. |  |
| Alzheimer Society of Ireland^7^ | 2013 | Ireland | Report | n.a. |  |
| Jacobsen^8^ | 2016 | Germany | Conference abstract | n.a. | n = 1 Focus groups |
| Lenz et al.^9^ | 2015 | Germany | Research Report | n.a. |  |
| Geschäftsstelle Demenzplan Schleswig-Holstein^10^ | 2016 | Germany | Policy document | n.a. |  |
| Keogh et al.^11^ | 2021 | Ireland | Research Report | n.a. | n = 1 Policy café |
| Keogh et al.^12^ | 2020 | Ireland | Conference abstract | n.a. |  |
| Neubauer et al^13^ | 2018 | Canada | Research report | n.a. | n = 1 Key stakeholder forum |
| Neubauer et al.^14^ | 2021 | Canada | Original study report | Multi Method Study | n = 1 Semi-structured individual interviews with confirmatory survey |
| Neubauer et al.^15^ | 2021 | Canada | Original study report | Survey | n = 1 Survey |
| Neubauer et al.^16^ | 2022 | Canada | Conference abstract | n.a. | n = 1 Focus groups |
| Shi et al.^17^ | 2022 | China | Original study report | Mixed-Methods Study | n = 1 Delphi |
| Littlejohn et al.^18^ | 2022 | UK, USA, France, Greece, Cyprus | Guideline report | n.a. | n = 1 Research user groups (RUGs) |
| Miah et al.^19^ | 2018 | UK | Study Protocol | Study Protocol for a qualitative study |  |
| Beattie et al.^20^ | 2021 | UK (Scotland) | Conference abstract | n.a. | n = 2 Engagement events  Online questionnaire |
| Scottish Government et al.^21^ | 2020 | UK (Scotland) | Government report | n.a. |  |
| Hare^22^ | 2016 | UK | Report | n.a. | n = 1 Group meeting with select committee members |
| Litherland^23^ | 2015 | UK | Report | n.a. |  |
| House of Lords^24^ | 2014 | UK | Government report | n.a. |  |
| Main et al.^25^ | 2023 | Canada | Conference abstract | n.a. | n = 1 Serving as members of working groups |
| Alzheimer Society Canada^26^ | 2023 | Canada | Guideline | n.a. |  |
| Beattie et al.^27^ | 2023 | UK (Scotland) | Conference abstract | n.a. | n = 4 Public consultation Engagement events Serving as members of working groups Consultation of the SDWG |
| Scottish Government^28^ | 2023 | UK (Scotland) | Government report | n.a. |  |
| Scottish Government^29^ | n.r. | UK (Scotland) | Government report | n.a. |  |
| Rankin^30^ | 2023 | UK (Scotland) | Conference abstract | n.a. |  |
| Alzheimer Scotland^31^ | n.r. | UK (Scotland) | Report | n.a. |  |
| Litherland^32^ | 2014 | UK | Conference abstract | n.a. | n = 4 Meetings with government ministers or Prime Minister Serving as members of working groups Individual interviews Focus groups |
| Mental Health Foundation United Kingdom^33^ | 2012 | UK | Report | n.a. |  |
| Weaks et al.^34^ | 2012 | UK | Report | n.a. |  |
| Alzheimer’s Society Northern Ireland^35^ | 2009 | UK | Report | n.a. |  |
| Moreno^36^ | 2023 | USA | Letter | n.a. | n = 2 Hearing with Social Security Administration (SSA) Listening session with medical association staff |
| Engedal et al.^37^ | 2015 | Norway | Conference abstract | n.a. | n = 1 Dialogue meetings |
| Goodenough et al.^38^ | 2022 | Australia | Report | n.a. | n = 3 Interviews and focus groups Online survey Serving as jury advisers |
| Australian Government Department of Health and Aged Care^39^ | 2023 | Australia | Government report | n.a. |  |
| Australian Government Department of Health and Aged Care^40^ | 2024 | Australia | Government report | n.a. |  |
| Sloan et al.^41^ | 2022 | UK (Scotland) | Conference abstract | n.a. | n = 4 Online engagement sessions Online survey Discussions and creative workshops (conference) Meeting with ministers |
| About Dementia et al.^42^ | 2021 | UK (Scotland) | Report | n.a. |  |
| About Dementia et al.^43^ | 2021 | UK (Scotland) | Report | n.a. |  |

**Abbreviations:** n.a.: not applicable; n.r.: not reported

References

1. Armstrong MJ, Mullins CD, Gronseth GS, Gagliardi AR. Impact of patient involvement on clinical practice guideline development: a parallel group study. *Implement Sci*. 2018;13(1):55. doi:10.1186/s13012-018-0745-6

2. Armstrong MJ, Gronseth GS, Day GS, Rheaume C, Alliance S, Mullins CD. Patient Stakeholder Versus Physician Preferences Regarding Amyloid PET Testing. *Alzheimer Dis Assoc Disord*. 2019;33(3):246-253. doi:10.1097/WAD.0000000000000311

3. Armstrong MJ, Gronseth GS, Gagliardi AR, Mullins CD. Participation and consultation engagement strategies have complementary roles: A case study of patient and public involvement in clinical practice guideline development. *Health Expect*. 2020;23(2):423-432. doi:10.1111/hex.13018

4. Begley E. Involving people with dementia in national policy development: A Case Study of the Irish National Dementia Strategy. *24^th^ Alzheimer Europe Conference, Glasgow, Scotland*. 2014. Accessed August 21, 2024.

5. Ireland Department of Health. *The Irish National Dementia Strategy*; 2014. Accessed August 21, 2024. <https://www.hse.ie/eng/dementia-pathways/files/the-irish-national-dementia-strategy.pdf>.

6. Alzheimer Society of Ireland. *Living with Dementia: Implications for the National Dementia Strategy: Summary of Roundtable Discussions*; 2013.

7. Alzheimer Society of Ireland. *National Strategy on Dementia: Summary of Consultation Process*; 2013. Accessed August 21, 2024. <https://www.lenus.ie/handle/10147/306069>.

8. Jacobsen W. Dementia Strategy Schleswig-Holstein. *26th Alzheimer Europe Conference, Copenhagen, Denmark*. 2016.

9. Lenz G, Micus-Loos C. *Rekonstruktion Der Angehörigen- Und Betroffenenperspektive Von Menschen Mit Demenz: Abschlussbericht Der Wissenschaftlichen Begleitung Des Demenzplan Schleswig-Holstein (Unpublished Report)*; 2015.

10. Geschäftsstelle Demenzplan Schleswig-Holstein. *Demenzplan Schleswig-Holstein: Anlagenband*; 2016.

11. Keogh F, Carney P, O’Shea E. Innovative methods for involving people with dementia and carers in the policymaking process. *Health Expect*. 2021;24(3):800-809. doi:10.1111/hex.13213

12. Keogh F, Irish Dementia Working Group, Whelan C. Developing guidelines for the involvement of people with dementia in policy, advisory, consultation and conference activities. *30^th^ Alzheimer Europe Conference, Online*. 2020.

13. Neubauer N, Hillier LM, Conway C, Beleno R, Liu L. Reflections of the use of locating technologies with persons with dementia: proceedings of a key stakeholder forum. *Neurodegener Dis Manag*. 2018;8(3):195-205. doi:10.2217/nmt-2018-0002

14. Neubauer NA, Liu L. Development and validation of a conceptual model and strategy adoption guidelines for persons with dementia at risk of getting lost. *Dementia (London)*. 2021;20(2):534-555. doi:10.1177/1471301219898350

15. Neubauer NA, Liu L. Dissemination and implementation of strategy adoption guidelines for persons with dementia at risk of getting lost. *Aging Ment Health*. 2021;25(3):528-534. doi:10.1080/13607863.2019.1699017

16. Neubauer NA, McLennan L, Leung E, Daum C, Zhang-Kennedy L, Liu L. An interactive guideline to mitigate the risks associated with getting lost among persons living with dementia. *13^th^ International Society for Gerontechnology World Conference, Daegu, Korea*. 2022. doi:10.4017/gt.2022.21.s.508.pp3

17. Shi C, Wong GHY, Choy JCP, Wong KKY, Lum TYS, Yu DSF. Are we on the same page? Multiple stakeholders and service users priorities for dementia care and policy: A Delphi study. *Int J Nurs Stud*. 2022;133. doi:10.1016/j.ijnurstu.2022.104300

18. Littlejohn J, Bowen M, Constantinidou F, et al. International Practice Recommendations for the Recognition and Management of Hearing and Vision Impairment in People with Dementia. *Gerontology*. 2022;68(2):121-135. doi:10.1159/000515892

19. Miah J, Dawes P, Leroi I, Parsons S, Starling B. A protocol to evaluate the impact of involvement of older people with dementia and age-related hearing and/or vision impairment in a multi-site European research study. *Res Involv Engagem*. 2018;4:44. doi:10.1186/s40900-018-0128-9

20. Beattie J, Berry D. Dementia and covid-19 – Scotland’s National Action Plan to continue to support recovery for people with dementia and their carers. *31^st^ Alzheimer Europe Conference, online*. 2021.

21. Scottish Government, Convention of Scottish Local Authorities. *Dementia and Covid-19 – National Action Plan to Continue to Support Recovery for People with Dementia and Their Carers*; 2020. Accessed August 21, 2024. <https://www.gov.scot/publications/dementia-covid-19-national-action-plan-continue-support-recovery-people-dementia-carers/>.

22. Hare P. Dementia without Walls: reflections on the Joseph Rowntree Foundation programme. *WWOP*. 2016;20(3):134-143. doi:10.1108/WWOP-06-2016-0012

23. Litherland R. *Developing a National User Movement of People with Dementia: Learning from the Dementia Engagement and Empowerment Project (DEEP)*; 2015. Accessed August 21, 2024. <https://www.jrf.org.uk/sites/default/files/migrated/files-research/developing_movement_dementia_summary.pdf>.

24. House of Lords. *Mental Capacity Act 2005: Committee Report*; 2014. Accessed August 21, 2024. <https://publications.parliament.uk/pa/ld201314/ldselect/ldmentalcap/139/139.pdf>.

25. Main S, Sivananthan S, Feldman S, et al. Canada’s First National Dementia Guidelines: A Collaborative Approach to Improving the Diagnosis Experience. *33^rd^ Alzheimer Europe Conference, Helsinki, Finland*. 2023.

26. Alzheimer Society Canada. *National Dementia Guidelines for Healthcare Providers: Disclosing and Communicating a Diagnosis of Dementia*; 2023. <https://alzheimer.ca/en/help-support/im-healthcare-provider/national-dementia-guidelines>.

27. Beattie J, Doherty R. Everyone’s Story: Scotland’s New National Dementia Strategy. *33^rd^ Alzheimer Europe Conference, Helsinki, Finland*. 2023.

28. Scottish Government. *A National Conversation to Inform a New Dementia Strategy for Scotland – What People Told Us*; 2023. Accessed August 21, 2024. <https://www.gov.scot/publications/national-conversation-inform-new-dementia-strategy-scotland-people-told/documents/>.

29. Scottish Government. *National Dementia Lived Experience Panel: The Story so Far (Unpublished Report)*; date unknown.

30. Rankin W. National Dementia Strategies - Ensuring meaningful engagement with those with Lived Experience. *33^rd^ Alzheimer Europe Conference, Helsinki, Finland*. 2023.

31. Alzheimer Scotland. *Public Engagement Response*; date unknown. Accessed August 21, 2024. <https://www.alzscot.org/sites/default/files/2022-12/Alzheimer%20Scotland%20Public%20Engagement%20Response%20-%20FINAL.pdf>.

32. Litherland R. Dementia Engagement & Empowerment Project – making involvement and influencing meaningful. *24^th^ Alzheimer Europe Conference, Glasgow, Scotland*. 2014.

33. Mental Health Foundation United Kingdom. *Ripple on the Pond: DEEP: The Engagement, Involvement and Empowerment of People with Dementia in Collective Influencing*; 2012.

34. Weaks D, Wilkinson H, Houston A, McKillop J. *Perspectives on Ageing with Dementia*; 2012. Accessed August 21, 2024. <https://www.jrf.org.uk/perspectives-on-ageing-with-dementia>.

35. Alzheimer’s Society Northern Ireland. *Listening Well: People with Dementia Informing Development of Health and Social Care Policy*; 2009.

36. Moreno M, Kline C, Shubeck E, Lanigan K, Fazio S. Engaging individuals living with dementia as stakeholders. *Alzheimers Dement (N Y)*. 2023;9(1). doi:10.1002/trc2.12366

37. Engedal K, Toft AK. Involving people with dementia in new dementia strategy. *25^th^ Alzheimer Europe Conference, Ljubljana, Slovenia*. 2015.

38. Goodenough B, Morris D. Improving accommodation in residential aged care. *Australian Journal of Dementia Care*. 2022;11(3).

39. Australian Government Department of Health and Aged Care. *Final Report on the Development of the Draft National Aged Care Design Principles and Guidelines*; 2023. Accessed August 21, 2024. <https://www.health.gov.au/resources/publications/draft-national-aged-care-design-principles-and-guidelines?language=en>.

40. Australian Government Department of Health and Aged Care. *Reimagining Where We Live: Jury Report*; 2024. Accessed August 21, 2024. <https://www.health.gov.au/sites/default/files/2024-04/jury-report-reimagining-where-we-live-design-ideas-competition.pdf>.

41. Sloan D, Meighan M, Manji K. “I don’t want things done to me, I want things done with me” – Engaging People Living with Dementia and Unpaid Carers in Responding to the Scottish Government’s National Care Service Consultation. *35^th^ Alzheimer’s Disease International Conference, London, United Kingdom*. 2022.

42. About Dementia, Age Scotland. *A National Care Service for Scotland: Consultation Overview*. Accessed August 21, 2024. <https://www.agescotland.org.uk/assets/000/000/684/15._national-care-service-consultation-briefing---pdf_original.pdf?1709819792>.

43. About Dementia, Age Scotland. *A National Care Service for Scotland: Consultation Response*; 2021. Accessed August 21, 2024. <https://www.agescotland.org.uk/assets/000/000/683/14._ncs---submitted-response---formatted-pdf_original.pdf?1709819729>.
