# Supplementary material for: Methods for Involving People With Dementia in Health Policy and Guideline Development: A Scoping Review
Source: Health Expect. 2025 Apr 3;28(2):e70250. doi: 10.1111/hex.70250 (PMC11968782; doi:10.1111/hex.70250)
Supplement: Supplementary file 4 — Supplement 4: Characteristics of participating people with dementia. [file HEX-28-e70250-s001.docx]

Supplement 4: Characteristics of participating people with dementia

|  |  | **Characteristics of participating people with dementia** | | | | | |  |  |
| --- | --- | --- | --- | --- | --- | --- | --- | --- | --- |
| **Involvement method** | **Reported target group** | **Number** | **Age** | **Sex** | **Dementia subtypes** | **Dementia stages** | **Recruitment** | **Participants besides people with dementia** | **Number of people with dementia in the total sample** |
| **Focus groups and interviews** | | | | | | | | | |
| Individual interviews^1^ | Not specified | 7 | 46-76 | n.r. | n.r. | Earlier stages | Alzheimer organisation | Exclusively people with dementia | 7/7 |
| Interviews and focus groups^2,3^ | Not specified | n.r. | n.r. | n.r. | n.r. | n.r. | n.r. | Carers and family members (n = n.r.) TOTAL n = n.r. | Unclear |
| Focus groups^4,5^ | Not specified | 4 | n.r. | 1 female 3 male | n.r. | n.r. | Pre-existing self-help group | 3 other focus groups with carers (n = 15) TOTAL n= 19 | 4/19 |
| Focus groups^6^ | Not specified | 7 | 60-80 | n.r. | n.r. | Earlier stages | n.r. | Exclusively people with dementia | 7/7 |
| Focus groups^1^ | Not specified | 7 | n.r. | 2 female 5 male | n.r. | Earlier stages | Alzheimer organisation | Informal carers also took part in the focus group (n = 10) TOTAL n = 17 | 7/17 |
| **Surveys and questionnaires** | | | | | | | | | |
| Survey^7^ | Lay persons | 29 | n.r. | n.r. | n.r. | n.r. | Alzheimer organisation | Clinicians, other patient representatives (n = 192) TOTAL n = 221 | 29/221 |
| Survey^8^ | Not specified | 7 | 65 (SD 11) | 2 female 5 male | n.r. | n.r. | Alzheimer organisation | Representatives from community organisations, family care-givers, paid caregivers, and other health professionals (n = 66) TOTAL n = 73 | 7/73 |
| Online survey^9,10^ | People in early stages of dementia with sufficient verbal skills | approx. 5 | n.r. | n.r. | n.r. | n.r. | Advertising and social media, publicly accessible | Open to other participants (n = n.r.) TOTAL n = 228 | Unclear |
| Online survey^2^ | Not specified | n.r. | n.r. | n.r. | n.r. | n.r. | n.r. | Older people, their families, carers (n = n.r.) TOTAL n = 734 | Unclear |
| Public consultation^11,12^ | Not specified | 7 | n.r. | n.r. | n.r. | n.r. | Informal contacts, through organisations (Alzheimer Scotland etc.), via word-of-mouth | Carers, professionals, other people with an interest in dementia, health and social care organisations (n = 160) TOTAL n = 167 | 7/167 |
| Online questionnaire^13,14^ | Not specified | n.r. | n.r. | n.r. | n.r. | n.r. | Pre-existing working group (National Dementia in Care Homes Working Group, National Dementia Strategy Implementation and Monitoring Group) | Informal carers, professionals, health and social care organisations.  TOTAL n = 99 | Unclear |
| Public comment^15^ | Lay persons | Unclear if people with dementia participated | n.r. | n.r. | n.r. | n.r. | n.r. | Physicians, researchers, anonymous participants (n = n.r.) TOTAL n = 54 | Unclear |
| **Public events** | | | | | | | | | |
| Engagement events^11,12,16^ | Not specified | n.r. | n.r. | n.r. | n.r. | n.r. | n.r. | Organisations and individuals, carers, health professionals, other people with an interest in dementia (n = n.r.) 110 engagement events in total TOTAL n = n.r. | Unclear |
| Engagement events^13,14^ | Not specified | n.r. | n.r. | n.r. | n.r. | n.r. | Pre-existing working group (National Dementia in Care Homes Working Group, National Dementia Strategy Implementation and Monitoring Group) | Informal carers, professionals, health and social care organisations.  TOTAL n > 100 | Unclear |
| Key stakeholder forum^17^ | Not specified | 3 | n.r. | n.r. | n.r. | n.r. | Alzheimer organisation | Consumers, care partners, Alzheimer Society representatives, social workers, healthcare professionals, technology industry representatives, researchers, government representatives (n = 106) TOTAL n = 109 | 3/109 |
| Dialogue Meetings^18^ | Not specified | n.r. | n.r. | n.r. | n.r. | n.r. | n.r. | Carers, employees of care organisations, health professionals (n = n.r.) TOTAL n ca. 700 | Unclear |
| Online Engagement Sessions^9,10^ | People in early stages of dementia with sufficient verbal skills | n.r. | n.r. | n.r. | n.r. | n.r. | Pre-existing groups, familiar persons and contacts, gatekeepers, snowballing | Exclusively people with dementia | Unclear |
| Discussions and creative workshops^9,10^ | People in early stages of dementia with sufficient verbal skills | approx. 20-30 | n.r. | n.r. | n.r. | n.r. | Pre-existing groups, familiar persons and contacts, gatekeepers, snowballing | n.r. | Unclear |
| **Meetings with decision makers** | | | | | | | | | |
| Group Meeting with Select Committee members^19-21^ | Not specified | 9 | n.r. | n.r. | n.r. | n.r. | Pre-existing self-help group (DEEP group) | Exclusively people with dementia | 9/9 |
| Meetings with government ministers or Prime Minister^22^ | Not specified | n.r. | n.r. | n.r. | n.r. | n.r. | Pre-existing working group (SDWG) | n.r. | Unclear |
| Meeting with minister^9,10^ | People in early stages of dementia with sufficient verbal skills | 2 | n.r. | n.r. | n.r. | n.r. | Previous contacts | Exclusively people with dementia | 2/2 |
| Hearing with Social Security Administration (SSA)^23^ | People in early dementia stages | 2 | 52 (male); <65 (female) | 1 female 1 male | Alzheimer (male) Mixed Alzheimer and vascular (female) | Earlier stages | Pre-existing working group (ESAG) | Care partners (n = 2) also took part in the hearing  TOTAL n = 4 | 2/4 |
| Listening session with medical association staff^23^ | People in early dementia stages | approx. 10-12 | n.r. | n.r. | n.r. | Earlier stages | Pre-existing working group (ESAG) | Care partners also took part (n = n.r.) TOTAL n = n.r. | Unclear |
| Roundtables^24^ | Not specified | 7 | n.r. | n.r. | n.r. | n.r. | Pre-existing working group (IDWG) | Carers, dementia representatives (n=10) TOTAL n = 17 | 7/17 |
| **Serving as members of working groups** | | | | | | | | | |
| Guideline drafting group^15^ | Lay persons | 1 | n.r. | n.r. | n.r. | MCI | Alzheimer organisation | Dementia representatives, physicians, guideline development specialists (n = 15) TOTAL n = 16 | 1/16 |
| Guideline development groups^25^ | Lay persons | 1 | n.r. | n.r. | n.r. | MCI | Alzheimer organisation | Dementia representatives, physicians, guideline development specialists (n = 15) TOTAL n = 16 | 1/16 |
| Serving as members of working groups^11,12^ | Not specified | 11 | n.r. | n.r. | n.r. | n.r. | Publicity and advertising | Carers of people with dementia (n = 11) TOTAL n = 22 | 11/22 |
| Serving as members of working groups^26,27^ | Not specified | 4 | n.r. | n.r. | Young-onset dementia of various types | Early-mid stages | n.r. | Care partners, physicians, health professionals were also part of the working groups (n = 23) TOTAL n = 27 | 4/27 |
| Serving as members of working groups^22^ | Not specified | 10 | n.r. | n.r. | n.r. | n.r. | Pre-existing working group (SDWG) | Yes, but details and number n.r. | Unclear |
| Research user groups (RUGs)^28,29^ | People with dementia with hearing and/or vision impairment | 9 | >65 | n.r. | Alzheimer's disease OR Mixed Dementia | Mild to moderate dementia, details n.r. | Local advertising through flyers, organisations, networks for older people/people with dementia | Informal carers were also part of the RUGs (n = 12) TOTAL n = 21 | 9/21 |
| Serving as jury advisers^2,30^ | Not specified | 6 | n.r. | 4 female 2 male | n.r. | n.r. | Alzheimer organisation | Exclusively people with dementia | 6/6  100% |
| Consultation with SDWG^16^ | Not specified | n.r. | n.r. | n.r. | n.r. | n.r. | Pre-existing working group (SDWG) | n.r. | Unclear |
| **Multiple-step methods** | | | | | | | | | |
| Semi-Structured individual interviews with confirmatory survey^31^ | People with mild to moderate dementia with MMSE > 10 | 5 | n.r. | n.r. | n.r. | mild dementia, details n.r. | Snowball sampling via key informants and Alzheimer organisations, recruitment from previous studies and authors' contacts | Family and formal caregivers, other persons experienced with wandering and dementia (n = 32) TOTAL n = 37 | 5/37 |
| Policy café^32^ | Not specified | 10 | n.r. | 5 female, 5 male | Young-onset and later-onset participants | n.r. | Pre-existing working group (IDWG) | Exclusively people with dementia | 10/10  100% |
| Delphi^33^ | Not specified | 45 | 79 (SD 19) | 35 female (77.8%); 10 male (22.2%) | n.r. | MCI: 16 (35.6%); mild dementia: 26 (57.8%); moderate dementia: 3 (6.7%) | Social care facilities | Exclusively people with dementia | 45/45  100% |

**Abbreviations**: ESAG: Early-Stage Advisory Group; SDWG: Scottish Dementia Working Group; IDWG: Irish Dementia Working Group; MMSE: Mini Mental State Examination; MCI: Mild cognitive impairment; n.r.: not reported

References

1. Alzheimer’s Society Northern Ireland. *Listening Well: People with Dementia Informing Development of Health and Social Care Policy*; 2009.

2. Goodenough B, Morris D. Improving accommodation in residential aged care. *Australian Journal of Dementia Care*. 2022;11(3).

3. Australian Government Department of Health and Aged Care. *Final Report on the Development of the Draft National Aged Care Design Principles and Guidelines*; 2023. Accessed August 21, 2024. <https://www.health.gov.au/resources/publications/draft-national-aged-care-design-principles-and-guidelines?language=en>.

4. Jacobsen W. Dementia Strategy Schleswig-Holstein. *26^th^ Alzheimer Europe Conference, Copenhagen, Denmark*. 2016.

5. Lenz G, Micus-Loos C. *Rekonstruktion Der Angehörigen- Und Betroffenenperspektive Von Menschen Mit Demenz: Abschlussbericht Der Wissenschaftlichen Begleitung Des Demenzplan Schleswig-Holstein (Unpublished Report)*; 2015.

6. Neubauer NA, McLennan L, Leung E, Daum C, Zhang-Kennedy L, Liu L. An interactive guideline to mitigate the risks associated with getting lost among persons living with dementia. *13^th^ International Society for Gerontechnology World Conference, Daegu, Korea*. 2022. doi:10.4017/gt.2022.21.s.508.pp3

7. Armstrong MJ, Gronseth GS, Day GS, Rheaume C, Alliance S, Mullins CD. Patient Stakeholder Versus Physician Preferences Regarding Amyloid PET Testing. *Alzheimer Dis Assoc Disord*. 2019;33(3):246-253. doi:10.1097/WAD.0000000000000311

8. Neubauer NA, Liu L. Dissemination and implementation of strategy adoption guidelines for persons with dementia at risk of getting lost. *Aging Ment Health*. 2021;25(3):528-534. doi:10.1080/13607863.2019.1699017

9. Sloan D, Meighan M, Manji K. “I don’t want things done to me, I want things done with me” – Engaging People Living with Dementia and Unpaid Carers in Responding to the Scottish Government’s National Care Service Consultation. *35^th^ Alzheimer’s Disease International Conference, London, United Kingdom*. 2022.

10. About Dementia, Age Scotland. *A National Care Service for Scotland: Consultation Response*; 2021. Accessed August 21, 2024. <https://www.agescotland.org.uk/assets/000/000/683/14._ncs---submitted-response---formatted-pdf_original.pdf?1709819729>.

11. Beattie J, Doherty R. Everyone’s Story: Scotland’s New National Dementia Strategy. *33^rd^ Alzheimer Europe Conference, Helsinki, Finland*. 2023.

12. Scottish Government. *A National Conversation to Inform a New Dementia Strategy for Scotland – What People Told Us*; 2023. Accessed August 21, 2024. <https://www.gov.scot/publications/national-conversation-inform-new-dementia-strategy-scotland-people-told/documents/>.

13. Beattie J, Berry D. Dementia and covid-19 – Scotland’s National Action Plan to continue to support recovery for people with dementia and their carers. *31^st^ Alzheimer Europe Conference, online*. 2021.

14. Scottish Government, Convention of Scottish Local Authorities. *Dementia and Covid-19 – National Action Plan to Continue to Support Recovery for People with Dementia and Their Carers*; 2020. Accessed August 21, 2024. <https://www.gov.scot/publications/dementia-covid-19-national-action-plan-continue-support-recovery-people-dementia-carers/>.

15. Armstrong MJ, Gronseth GS, Gagliardi AR, Mullins CD. Participation and consultation engagement strategies have complementary roles: A case study of patient and public involvement in clinical practice guideline development. *Health Expect*. 2020;23(2):423-432. doi:10.1111/hex.13018

16. Rankin W. National Dementia Strategies - Ensuring meaningful engagement with those with Lived Experience. *33^rd^ Alzheimer Europe Conference, Helsinki, Finland*. 2023.

17. Neubauer N, Hillier LM, Conway C, Beleno R, Liu L. Reflections of the use of locating technologies with persons with dementia: proceedings of a key stakeholder forum. *Neurodegener Dis Manag*. 2018;8(3):195-205. doi:10.2217/nmt-2018-0002

18. Engedal K, Toft AK. Involving people with dementia in new dementia strategy. *25^th^ Alzheimer Europe Conference, Ljubljana, Slovenia*. 2015.

19. Litherland R. *Developing a National User Movement of People with Dementia: Learning from the Dementia Engagement and Empowerment Project (DEEP)*; 2015. Accessed August 21, 2024. <https://www.jrf.org.uk/sites/default/files/migrated/files-research/developing_movement_dementia_summary.pdf>.

20. Hare P. Dementia without Walls: reflections on the Joseph Rowntree Foundation programme. *WWOP*. 2016;20(3):134-143. doi:10.1108/WWOP-06-2016-0012

21. House of Lords. *Mental Capacity Act 2005: Committee Report*; 2014. Accessed August 21, 2024. <https://publications.parliament.uk/pa/ld201314/ldselect/ldmentalcap/139/139.pdf>.

22. Weaks D, Wilkinson H, Houston A, McKillop J. *Perspectives on Ageing with Dementia*; 2012. Accessed August 21, 2024. <https://www.jrf.org.uk/perspectives-on-ageing-with-dementia>.

23. Moreno M, Kline C, Shubeck E, Lanigan K, Fazio S. Engaging individuals living with dementia as stakeholders. *Alzheimers Dement (N Y)*. 2023;9(1). doi:10.1002/trc2.12366

24. Begley E. Involving people with dementia in national policy development: A Case Study of the Irish National Dementia Strategy. *24^th^ Alzheimer Europe Conference, Glasgow, Scotland*. 2014. Accessed August 21, 2024.

25. Armstrong MJ, Mullins CD, Gronseth GS, Gagliardi AR. Impact of patient involvement on clinical practice guideline development: a parallel group study. *Implement Sci*. 2018;13(1):55. doi:10.1186/s13012-018-0745-6

26. Main S, Sivananthan S, Feldman S, et al. Canada’s First National Dementia Guidelines: A Collaborative Approach to Improving the Diagnosis Experience. *33^rd^ Alzheimer Europe Conference, Helsinki, Finland*. 2023.

27. Alzheimer Society Canada. *National Dementia Guidelines for Healthcare Providers: Disclosing and Communicating a Diagnosis of Dementia*; 2023. <https://alzheimer.ca/en/help-support/im-healthcare-provider/national-dementia-guidelines>.

28. Littlejohn J, Bowen M, Constantinidou F, et al. International Practice Recommendations for the Recognition and Management of Hearing and Vision Impairment in People with Dementia. *Gerontology*. 2022;68(2):121-135. doi:10.1159/000515892

29. Miah J, Dawes P, Leroi I, Parsons S, Starling B. A protocol to evaluate the impact of involvement of older people with dementia and age-related hearing and/or vision impairment in a multi-site European research study. *Res Involv Engagem*. 2018;4:44. doi:10.1186/s40900-018-0128-9

30. Australian Government Department of Health and Aged Care. *Reimagining Where We Live: Jury Report*; 2024. Accessed August 21, 2024. <https://www.health.gov.au/sites/default/files/2024-04/jury-report-reimagining-where-we-live-design-ideas-competition.pdf>.

31. Neubauer NA, Liu L. Development and validation of a conceptual model and strategy adoption guidelines for persons with dementia at risk of getting lost. *Dementia (London)*. 2021;20(2):534-555. doi:10.1177/1471301219898350

32. Keogh F, Carney P, O’Shea E. Innovative methods for involving people with dementia and carers in the policymaking process. *Health Expect*. 2021;24(3):800-809. doi:10.1111/hex.13213

33. Shi C, Wong GHY, Choy JCP, Wong KKY, Lum TYS, Yu DSF. Are we on the same page? Multiple stakeholders and service users priorities for dementia care and policy: A Delphi study. *Int J Nurs Stud*. 2022;133. doi:10.1016/j.ijnurstu.2022.104300
