# Supplementary material for: Methods for Involving People With Dementia in Health Policy and Guideline Development: A Scoping Review
Source: Health Expect. 2025 Apr 3;28(2):e70250. doi: 10.1111/hex.70250 (PMC11968782; doi:10.1111/hex.70250)
Supplement: Supplementary file 5 — Supplement 5: Analysis of involvement mechanisms according to the public engagement typology by Rowe and Frewer1. [file HEX-28-e70250-s002.docx]

Supplement 5: Analysis of involvement mechanisms according to the public engagement typology by Rowe and Frewer^1^

| **Involvement method** | **Class of public engagement** | **Participant selection** | **Facilitated elicitation** | **Response mode** | **Information input** | **Information transfer** | **Structured aggregation** |
| --- | --- | --- | --- | --- | --- | --- | --- |
| **Focus groups and interviews** | | | | | | | |
| Individual interviews^2^ | Consultation | Controlled | Facilitated | Open | n.a. | FTF | Unstructured |
| Interviews and focus groups^3,4^ | Consultation | Controlled | Facilitated | Open | n.a. | FTF | Unstructured |
| Focus groups^5,6^ | Consultation | Controlled | Facilitated | Open | n.a. | FTF | Unstructured |
| Focus groups^7^ | Consultation | Controlled | Facilitated | Open | n.a. | FTF | Unstructured |
| Focus groups^2^ | Consultation | Controlled | Facilitated | Open | n.a. | FTF | Unstructured |
| **Surveys and questionnaires** | | | | | | | |
| Survey^8^ | Consultation | Uncontrolled | Not facilitated | Closed | n.a. | Non-FTF | Structured |
| Survey^9^ | Consultation | Controlled | Not facilitated | Closed | n.a. | Non-FTF | Structured |
| Online survey^10,11^ | Consultation | Uncontrolled | Not facilitated | Open and Closed | n.a. | Non-FTF | Structured |
| Online survey^3^ | Consultation | Uncontrolled | Not facilitated | Unclear | n.a. | Non-FTF | Unclear |
| Public consultation^12,13^ | Consultation | Uncontrolled | Not facilitated | Open | n.a. | Non-FTF | Unstructured |
| Online questionnaire^14,15^ | Consultation | Unclear | Not facilitated | Unclear | n.a. | Non-FTF | Unclear |
| Public comment^16^ | Consultation | Uncontrolled | Not facilitated | Open | n.a. | Non-FTF | Unclear |
| **Public events** | | | | | | | |
| Engagement events^12,13,17^ | Consultation | Uncontrolled | Facilitated | Open | n.a. | FTF | Unstructured |
| Engagement events^14,15^ | Consultation | Unclear | Facilitated | Open | n.a. | FTF | Unclear |
| Key stakeholder forum^18^ | Participation | Controlled | Facilitated | Open | Flexible | FTF | Unstructured |
| Dialogue Meetings^19^ | Consultation | Uncontrolled | Facilitated | Open | n.a. | FTF | Unstructured |
| Online Engagement Sessions^10,11^ | Consultation | Controlled | Facilitated | Open | n.a. | FTF | Unclear |
| Discussions and creative workshops^10,11^ | Consultation | Controlled | Facilitated | Open | n.a. | FTF | Unclear |
| **Meeting with decision makers** | | | | | | | |
| Group Meeting with Select Committee members^20-22^ | Consultation | Controlled | Facilitated | Open | n.a. | FTF | Unstructured |
| Meetings with government ministers or Prime Minister^23^ | Consultation | Controlled | Facilitated | Open | n.a. | FTF | Unstructured |
| Meeting with minister^10,11^ | Consultation | Controlled | Facilitated | Open | n.a. | FTF | Unclear |
| Hearing with Social Security Administration (SSA)^24^ | Consultation | Controlled | Facilitated | Open | n.a. | FTF | Unstructured |
| Listening session with medical association staff^24^ | Consultation | Controlled | Facilitated | Open | n.a. | FTF | Unstructured |
| Roundtables^25^ | Consultation | Controlled | Facilitated | Open | n.a. | FTF | Unstructured |
| **Serving as members of working groups** | | | | | | | |
| Guideline drafting group^16^ | Participation | Controlled | Facilitated | Open | Flexible | FTF | Structured |
| Guideline development groups^26^ | Participation | Controlled | Facilitated | Open | Flexible | FTF | Structured |
| Serving as members of working groups^12,13^ | Participation | Controlled | Unclear | Unclear | Unclear | Unclear | Unclear |
| Serving as members of working groups^27,28^ | Participation | Controlled | Facilitated | Open | Flexible | FTF | Structured |
| Serving as members of working groups^23^ | Participation | Controlled | Facilitated | Open | Flexible | FTF | Unclear |
| Research user groups (RUGs)^29,30^ | Consultation | Controlled | Facilitated | Open | n.a. | FTF | Unstructured |
| Serving as jury advisers^3,31^ | Participation | Controlled | Facilitated | Open | Flexible | FTF | Unstructured |
| Consultation with SDWG^17^ | Consultation | Controlled | Facilitated | Open | n.a. | FTF | Unstructured |
| **Multiple-step methods** | | | | | | | |
| Semi-Structured individual interviews with confirmatory survey^32^ | Consultation | Controlled | Facilitated | Open and Closed | n.a. | FTF and Non-FTF | Structured |
| Policy café^33^ | Consultation | Controlled | Facilitated | Open | n.a. | FTF | Structured |
| Delphi^34^ | Consultation | Controlled | Facilitated | Open and closed | n.a. | Non-FTF | Structured |

**Abbreviations:** FTF: face-to-face; SDWG: Scottish Dementia Working Group; n.a.: not applicable

References

1. Rowe G, Frewer LJ. A Typology of Public Engagement Mechanisms. *Science, Technology, & Human Values*. 2005;30(2):251-290. doi:10.1177/0162243904271724

2. Alzheimer’s Society Northern Ireland. *Listening Well: People with Dementia Informing Development of Health and Social Care Policy*; 2009.

3. Goodenough B, Morris D. Improving accommodation in residential aged care. *Australian Journal of Dementia Care*. 2022;11(3).

4. Australian Government Department of Health and Aged Care. *Final Report on the Development of the Draft National Aged Care Design Principles and Guidelines*; 2023. Accessed August 21, 2024. <https://www.health.gov.au/resources/publications/draft-national-aged-care-design-principles-and-guidelines?language=en>.

5. Jacobsen W. Dementia Strategy Schleswig-Holstein. *26^th^ Alzheimer Europe Conference, Copenhagen, Denmark*. 2016.

6. Lenz G, Micus-Loos C. *Rekonstruktion Der Angehörigen- Und Betroffenenperspektive Von Menschen Mit Demenz: Abschlussbericht Der Wissenschaftlichen Begleitung Des Demenzplan Schleswig-Holstein (Unpublished Report)*; 2015.

7. Neubauer NA, McLennan L, Leung E, Daum C, Zhang-Kennedy L, Liu L. An interactive guideline to mitigate the risks associated with getting lost among persons living with dementia. *13^th^ International Society for Gerontechnology World Conference, Daegu, Korea*. 2022. doi:10.4017/gt.2022.21.s.508.pp3

8. Armstrong MJ, Gronseth GS, Day GS, Rheaume C, Alliance S, Mullins CD. Patient Stakeholder Versus Physician Preferences Regarding Amyloid PET Testing. *Alzheimer Dis Assoc Disord*. 2019;33(3):246-253. doi:10.1097/WAD.0000000000000311

9. Neubauer NA, Liu L. Dissemination and implementation of strategy adoption guidelines for persons with dementia at risk of getting lost. *Aging Ment Health*. 2021;25(3):528-534. doi:10.1080/13607863.2019.1699017

10. Sloan D, Meighan M, Manji K. “I don’t want things done to me, I want things done with me” – Engaging People Living with Dementia and Unpaid Carers in Responding to the Scottish Government’s National Care Service Consultation. *35^th^ Alzheimer’s Disease International Conference, London, United Kingdom*. 2022.

11. About Dementia, Age Scotland. *A National Care Service for Scotland: Consultation Response*; 2021. Accessed August 21, 2024. <https://www.agescotland.org.uk/assets/000/000/683/14._ncs---submitted-response---formatted-pdf_original.pdf?1709819729>.

12. Beattie J, Doherty R. Everyone’s Story: Scotland’s New National Dementia Strategy. *33^rd^ Alzheimer Europe Conference, Helsinki, Finland*. 2023.

13. Scottish Government. *A National Conversation to Inform a New Dementia Strategy for Scotland – What People Told Us*; 2023. Accessed August 21, 2024. <https://www.gov.scot/publications/national-conversation-inform-new-dementia-strategy-scotland-people-told/documents/>.

14. Beattie J, Berry D. Dementia and covid-19 – Scotland’s National Action Plan to continue to support recovery for people with dementia and their carers. *31^st^ Alzheimer Europe Conference, online*. 2021.

15. Scottish Government, Convention of Scottish Local Authorities. *Dementia and Covid-19 – National Action Plan to Continue to Support Recovery for People with Dementia and Their Carers*; 2020. Accessed August 21, 2024. <https://www.gov.scot/publications/dementia-covid-19-national-action-plan-continue-support-recovery-people-dementia-carers/>.

16. Armstrong MJ, Gronseth GS, Gagliardi AR, Mullins CD. Participation and consultation engagement strategies have complementary roles: A case study of patient and public involvement in clinical practice guideline development. *Health Expect*. 2020;23(2):423-432. doi:10.1111/hex.13018

17. Rankin W. National Dementia Strategies - Ensuring meaningful engagement with those with Lived Experience. *33^rd^ Alzheimer Europe Conference, Helsinki, Finland*. 2023.

18. Neubauer N, Hillier LM, Conway C, Beleno R, Liu L. Reflections of the use of locating technologies with persons with dementia: proceedings of a key stakeholder forum. *Neurodegener Dis Manag*. 2018;8(3):195-205. doi:10.2217/nmt-2018-0002

19. Engedal K, Toft AK. Involving people with dementia in new dementia strategy. *25^th^ Alzheimer Europe Conference, Ljubljana, Slovenia*. 2015.

20. Litherland R. *Developing a National User Movement of People with Dementia: Learning from the Dementia Engagement and Empowerment Project (DEEP)*; 2015. Accessed August 21, 2024. <https://www.jrf.org.uk/sites/default/files/migrated/files-research/developing_movement_dementia_summary.pdf>.

21. Hare P. Dementia without Walls: reflections on the Joseph Rowntree Foundation programme. *WWOP*. 2016;20(3):134-143. doi:10.1108/WWOP-06-2016-0012

22. House of Lords. *Mental Capacity Act 2005: Committee Report*; 2014. Accessed August 21, 2024. <https://publications.parliament.uk/pa/ld201314/ldselect/ldmentalcap/139/139.pdf>.

23. Weaks D, Wilkinson H, Houston A, McKillop J. *Perspectives on Ageing with Dementia*; 2012. Accessed August 21, 2024. <https://www.jrf.org.uk/perspectives-on-ageing-with-dementia>.

24. Moreno M, Kline C, Shubeck E, Lanigan K, Fazio S. Engaging individuals living with dementia as stakeholders. *Alzheimers Dement (N Y)*. 2023;9(1). doi:10.1002/trc2.12366

25. Begley E. Involving people with dementia in national policy development: A Case Study of the Irish National Dementia Strategy. *24^th^ Alzheimer Europe Conference, Glasgow, Scotland*. 2014. Accessed August 21, 2024.

26. Armstrong MJ, Mullins CD, Gronseth GS, Gagliardi AR. Impact of patient involvement on clinical practice guideline development: a parallel group study. *Implement Sci*. 2018;13(1):55. doi:10.1186/s13012-018-0745-6

27. Main S, Sivananthan S, Feldman S, et al. Canada’s First National Dementia Guidelines: A Collaborative Approach to Improving the Diagnosis Experience. *33^rd^ Alzheimer Europe Conference, Helsinki, Finland*. 2023.

28. Alzheimer Society Canada. *National Dementia Guidelines for Healthcare Providers: Disclosing and Communicating a Diagnosis of Dementia*; 2023. <https://alzheimer.ca/en/help-support/im-healthcare-provider/national-dementia-guidelines>.

29. Littlejohn J, Bowen M, Constantinidou F, et al. International Practice Recommendations for the Recognition and Management of Hearing and Vision Impairment in People with Dementia. *Gerontology*. 2022;68(2):121-135. doi:10.1159/000515892

30. Miah J, Dawes P, Leroi I, Parsons S, Starling B. A protocol to evaluate the impact of involvement of older people with dementia and age-related hearing and/or vision impairment in a multi-site European research study. *Res Involv Engagem*. 2018;4:44. doi:10.1186/s40900-018-0128-9

31. Australian Government Department of Health and Aged Care. *Reimagining Where We Live: Jury Report*; 2024. Accessed August 21, 2024. <https://www.health.gov.au/sites/default/files/2024-04/jury-report-reimagining-where-we-live-design-ideas-competition.pdf>.

32. Neubauer NA, Liu L. Development and validation of a conceptual model and strategy adoption guidelines for persons with dementia at risk of getting lost. *Dementia (London)*. 2021;20(2):534-555. doi:10.1177/1471301219898350

33. Keogh F, Carney P, O’Shea E. Innovative methods for involving people with dementia and carers in the policymaking process. *Health Expect*. 2021;24(3):800-809. doi:10.1111/hex.13213

34. Shi C, Wong GHY, Choy JCP, Wong KKY, Lum TYS, Yu DSF. Are we on the same page? Multiple stakeholders and service users priorities for dementia care and policy: A Delphi study. *Int J Nurs Stud*. 2022;133. doi:10.1016/j.ijnurstu.2022.104300
